# Measuring perceived fitness interdependence between humans and non-humans

**DOI:** 10.1017/ehs.2024.10

**Published:** 2024-02-27

**Authors:** Katie Lee, Darragh Hare, Bernd Blossey

**Affiliations:** 1 Department of Natural Resources and the Environment, Cornell University, Ithaca, NY, USA; 2 Wildlife Conservation Research Unit, Department of Biology, University of Oxford, Oxford, UK

**Keywords:** Conservation ethics, cooperation, evolution of morality, perceived fitness interdependence

## Abstract

Conservation ethics (i.e. moral concern for non-human organisms) are widespread, but we lack a comprehensive explanation for why people care about other species at all, and why they express strong moral concern for some species but not others. Recent theory suggests that conservation ethics might be rooted in cooperation between humans and members of other species. Building on central predictions of this eco-evolutionary theory, we conducted an online study (*N* = 651) and exploratory factor analysis to develop two scales that independently measure perceived fitness interdependence (PFI) and conservation ethics. The PFI scale measures perceived shared fate as a proximate indicator of human fitness interdependence with non-human organisms (i.e. the degree to which humans and other organisms influence each other's evolutionary success, that is, survival and reproduction). We designed the conservation ethics scale to measure moral beliefs and attitudes regarding those organisms. Both scales are composed of two factors and demonstrate good internal reliability. By combining insights from various branches of the evolutionary human sciences, including evolutionary anthropology, evolutionary psychology and human behavioural ecology, we offer empirical tools to investigate eco-evolutionary foundations of conservation ethics and behaviour.

## Graphical abstract

**Figure:**
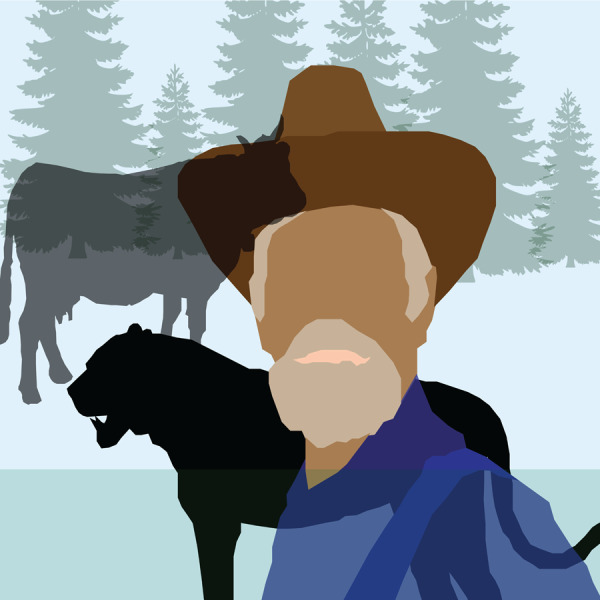

**Social media summary:** New scales shed light on eco-evolutionary explanations for human morality and cooperation with other species.

## Introduction

There is fundamental uncertainty as to why people care about wild species at all and why they feel strong moral obligations towards some species but not others (Chan et al., 2016; Hare et al., 2018; Lehnen et al., 2022; Soulé, 2013). Nevertheless, concerns about effects of human exploitation of other species are reflected in value systems (de Groot et al., 2011; Gamborg & Jensen, 2016; Teel & Manfredo, 2010; Teel et al., 2010) and cultural norms worldwide (Artelle et al., 2018; Berkes, 2017; Turner et al., 2000, 2009). Despite contradictory views about the role that human interests should play (compared with non-human interests) in conservation objectives (Hare et al., 2018), the widespread concern about other species’ well-being in value systems and cultural norms across societies suggests that humans express moral responsibility towards other species. Global biodiversity declines and contentions about how conservation efforts should be allocated call for an interdisciplinary approach to understanding *conservation ethics* (italic terms are defined in the glossary in Box 1). Despite recent work showing an evolutionary cooperative foundation for morality among people, specific linkages between evolution, morality and conservation have not been explored. An eco-evolutionary framework for understanding conservation ethics could provide novel insights into the *adaptive* mechanism underlying cooperation with other species and propose an ultimate explanation for why conservation ethics vary (Hare et al., 2018).

Box 1. Glossary.

**Table:** 

Term	Definition
Conservation ethics Adaptive Intrinsic value Instrumental value Relational value Cooperation Inclusive fitness Moral standing Emotional shared fate Domination Mutualism Anthropocentrism Zoocentrism Biocentrism Ecocentrism Moral inclusivity	Moral concern regarding members of wild species Behaviors that ultimately increase the inclusive fitness of individuals engaged in those behaviours and are favoured by natural selection (Hamilton, 1964) The inherent value attributed to organisms that is over and above their instrumental or utilitarian value to humans (Callicott, 1979; Lute et al., 2016; Vucetich et al., 2015) The value attributed to organisms derived from benefits they provide to humans (Justus et al., 2009) The value attributed to organisms derived from relationships and responsibilities to them (Chan et al., 2016) Interactions between individuals that provide inclusive fitness benefits to all individuals engaged in those interactions (Axelrod & Hamilton, 1981; Bowles & Gintis, 2011; Hamilton, 1964) Sum of an individual's personal fitness and the fitness of all its relatives, weighted by genetic relatedness (Hamilton, 1964) Moral concern for organisms deemed worthy of moral consideration (Crimston et al., 2016; Piazza et al., 2014; Singer, 2011) An individual's affective response to changes in the well-being of interdependent organisms (Ayers et al., 2023) Orientation towards wildlife that promotes human well-being over the well-being of other species, justifies treatment of wildlife in utilitarian terms and considers actions harming wildlife for human benefit as more permissible (Teel & Manfredo, 2010) Orientation towards wildlife that promotes relationships of trust with humans, perceives wildlife as deserving of rights and care, and considers actions harming wildlife for human benefit as less permissible (Teel & Manfredo, 2010) View of attributing intrinsic value only to humans, such that only humans are included in one's moral community (Baxter, 1974; Vucetich et al., 2015) View of attributing intrinsic value to humans and some members of wild species, often based on criteria such as sentience (Lute et al., 2016; Singer, 2011) View of attributing intrinsic value to all organisms (Taylor, 1981; Vucetich et al., 2015) View of attributing intrinsic value to all life forms, including individual organisms and ecological collectives, such as populations, species and ecosystems (Johnson, 1992; Vucetich et al., 2015) The inclusion of organisms possessing intrinsic value in one's moral community, such that anthropocentrism is the least inclusive and ecocentrism is the most inclusive (Batavia et al., 2020)

Conservation efforts are typically morally justified using frameworks of *intrinsic* (Lute et al., 2016; Vucetich et al., 2015), *instrumental* (Justus et al., 2009) or *relational value* (Arias-Arévalo et al., 2017; Chan et al., 2016), despite recognition that such value orientations cannot capture the full diversity of conservation ethics (Chan et al., 2016; Sandbrook et al., 2011). Although justifications for conservation can be scrutinised in terms of these ethical frameworks, evolutionary scientists have yet to explain why people care about other species at all (i.e. the ultimate adaptive reason for *cooperation*). The evolutionary theory of morality-as-cooperation (MAC) proposes that morality evolves to solve cooperation problems and can explain moral behaviour among humans (Curry, 2016; Curry et al., 2020; Curry et al., 2019; Tomasello & Vaish, 2013). Empirical tests of MAC also demonstrate that people's moral psychology (i.e. proximate mechanisms) maps onto this evolutionary cooperative theory. In fact, cooperation between species could explain why people express moral concern for non-human organisms, why they express greater concern for some organisms than others, and why this concern for different organisms varies across diverse ecological and socio-cultural contexts (Hare et al., 2018). However, whether MAC's fundamental logic applies to a conservation context has yet to be empirically tested.

Although cooperation apparently contradicts Darwin's influential idea of ‘nature red in tooth and claw’ (Bowles & Gintis, 2011; Darwin, 1859), cooperation is a critical component of social behaviour in many species, from microbes to plants to people. Natural selection should favour behaviour that benefits one's own reproductive success or that of genetically related individuals (i.e. *inclusive fitness*; Hamilton, 1964). Yet interspecific cooperation is widespread (Kiers et al., 2011) and manifests across all levels of ecological organisation (Barker et al., 2017; Harcombe, 2010; Sachs et al., 2004; West et al., 2007). However, ultimate justifications for cooperation, including inclusive fitness (Hamilton, 1964), reciprocal altruism (Axelrod & Hamilton, 1981; Trivers, 1971) and stakeholder theory (Roberts, 2005), constitute a rich body of work on what motivates cooperation and determines who individuals should cooperate with. Hamilton demonstrated that natural selection would favour cooperating with kin over non-kin by virtue of shared genes (Hamilton, 1964). Nevertheless, cooperation amongst unrelated individuals is widespread and prevalent in our everyday lives, from cooperating with peers to helping strangers even without expectation of reciprocation (Fehr & Fischbacher, 2003; Roberts, 2005).

**Figure 1.**
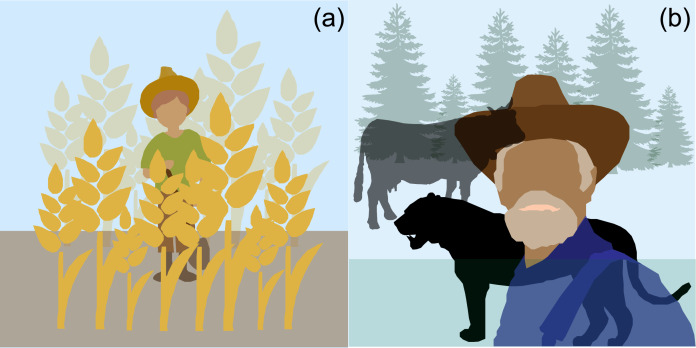
Examples of positive and negative fitness interdependence between humans and wildlife. Interdependence with non-human species is ubiquitous in human societies and refers to the degree of (a) positive or (b) negative influence of individuals’ outcomes on one another's fitness and well-being (Aktipis et al., 2018). (a) Most wheat is cultivated by farmers whose livelihoods depend on their wheat crop. This dependence on agriculture for livelihoods makes it more likely that farmers will care for their crops and optimize local growing conditions to increase yield. (b) In India, people who live in close proximity to large carnivores, such as tigers (*Panthera tigris*) and leopards (*Panthera pardus*), are more at risk and therefore negatively affected by livestock predation (e.g. cows, buffalos) than people who live further away (Ramesh et al., 2020). Thus, a prediction of conservation ethics based on fitness interdependence is that livestock farmers would express lower moral concern for predators than crop farmers who benefit from the predation of animals responsible for crop depredation.

A powerful explanation for non-kin cooperation rooted in evolutionary logic is fitness interdependence: ‘the degree to which two or more organisms positively or negatively influence each other's success in replicating their genes’ (Aktipis et al., 2018: 429). Fitness interdependence among individuals, including individuals who are not genetically related, can arise from shared fates and interests established and enhanced by socio-cultural norms and institutions (Aktipis et al., 2011, 2018). Fitness interdependence can be positive, as when interacting individuals increase each other's fitness (e.g. symbiosis or mutualism), and negative, as when interacting individuals compete for the same limited resources (e.g. predation or parasitism), or neutral (Ayers et al., 2023; Cronk et al., 2019) (Figure 1). However, it is impossible in any given situation for an individual to know definitively their degree of fitness interdependence with others (Ayers et al., 2023). The perceived fitness interdependence (PFI) scale captures underlying proximate aspects of interdependent relationships by assessing the degree to which an individual's emotional and fitness outcomes intertwine with potential outcomes of specific others (Ayers et al., 2023). Because proximal indicators of aligned fates, such as ‘closeness’ and ‘oneness’ are considered cues to infer interdependence and long-term fitness outcomes (Balliet et al., 2017; Columbus et al., 2020; Gerpott et al., 2018; Korchmaros & Kenny, 2001), fitness interdependence can be approximated using measures of these variables.

The PFI scale has hitherto only been applied to human–human cooperation. Yet its strong correlation with other broad measures of interdependence, such as welfare tradeoff ratios (Delton & Robertson, 2016; Sznycer et al., 2019; Tooby et al., 2008) and willingness to help others even in the absence of reciprocity (Roberts, 2005), makes it a plausible framework for assessing cooperation between humans and non-humans. Using eco-evolutionary models, Hare et al. (2018) extend the fundamental logic of fitness interdependence to include interdependence between humans and other species. Specifically, Hare et al.'s (2018) models illustrated the possibility that conservation ethics evolve because people's fitness covaries with the success of other species. For all individuals, the strength and the sign (positive or negative) of this covariance will vary with different species. Hare et al. (2018) predict that individuals will care most about species with the strongest positive fitness interdependence and express antipathy towards species with the strongest negative fitness interdependence. However, no study has yet empirically tested predictions from these models. Although social and environmental psychologists have explored many proximate mechanisms for conservation behaviours including attitudes, beliefs, values and norms, the evolutionary bases of such behaviours have not been extensively investigated (van Vugt et al., 2014). Bridging ultimate and proximate explanations requires this important knowledge gap to be filled.

**Figure 2.**
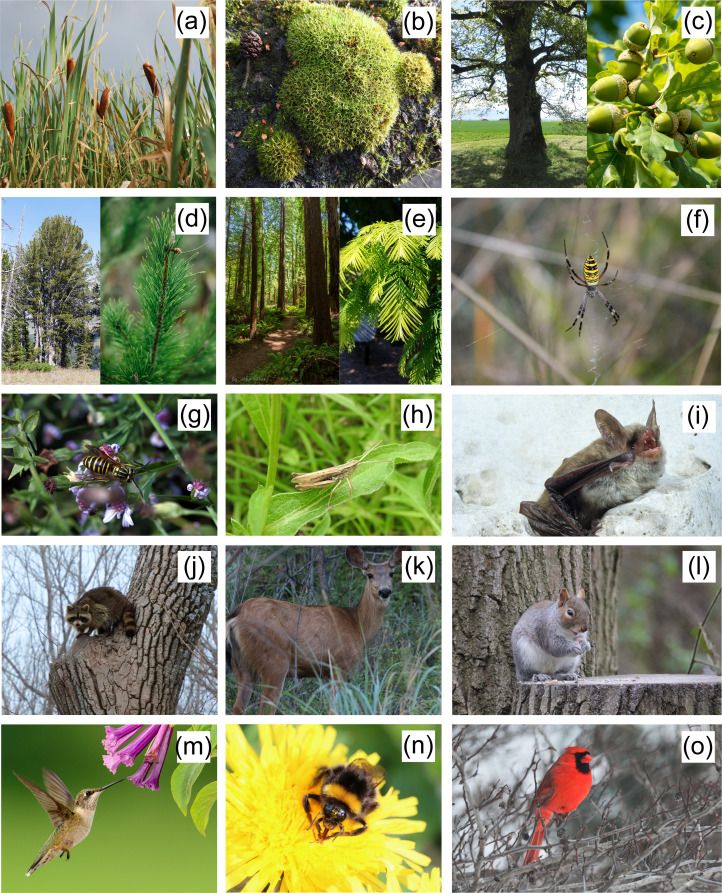
Target organisms grouped by species type (a–e, plants; f–j, nasty animals; k–o, nice animals): (a) cattail, (b) moss, (c) oak, (d) pine, (e) redwood, (f) spider, (g) yellowjacket, (h) grasshopper, (i) bat, (j) raccoon, (k) deer, (l) squirrel, (m) hummingbird, (n) bumblebee and (o) cardinal. We sourced all images with unrestricted use allowed on Flickr (www.flickr.com). Photo credits: (a) USDA NRCS Montana; (b) Rob Mitchell; (c) paulmacwhirr, John K Thorne; (d) Yellowstone National Park, James; (e) John Fisher, Dan Keck; (f) Alejandro Gómez Vilches; (g) Insects Unlocked; (h) Carrie Stephens; (i) Land Between the Lakes KY/TN; (j) USFWS Midwest Region; (k) Dominic Bordin; (l) Wildlife Terry; (m) Maria Elenilda Souza; (n) Wildlife Terry; and (o) USFWS Midwest Region.

Our objectives are to develop new scales for (1) human–non-human PFI and (2) conservation ethics as the necessary first steps to testing these predictions. Our work expands on related but separate aspects of evolved moral psychology derived from published measures that index fitness interdependence among humans (Ayers et al., 2023) and *moral standing* regarding other humans and non-humans (Berndsen & van der Pligt, 2005; Crimston et al., 2016; Graham et al., 2011; Piazza et al., 2014). To this end, we devised an online study using 15 wild plant and animal species as target organisms (Figure 2). We develop these scales independently to provide the necessary tools for future investigation on the theoretical possibility that the perceived relationship of other species to human fitness (i.e. PFI) might predict conservation ethics towards those species.

## Methods

### Item generation

We generated items for each scale focusing on indicators of (1) how people view their well-being as associated with the well-being of other species (i.e. PFI) and (2) their moral concern for those species (i.e. conservation ethics) (see Table 1 for all items that were considered). We either adapted or used unchanged nine of 25 PFI items from a recent scale assessing perceived and emotional shared fate among humans (Ayers et al., 2023). We adapted the items by modifying their wording to be used in the context of human interaction with other species. We adapted or used unchanged all conservation ethics items except for one (i.e. people should deny moral concern for [target]) from published measures of moral standing regarding other humans and non-humans as no single validated measure of conservation ethics existed. We derived one item from Berndsen and van der Pligt (2005), one item from Crimston et al. (2016), two items from Graham et al. (2011) and six items from Piazza et al. (2014). All authors were involved in item creation and provided feedback on the appropriateness of these items in the context of human–non-human relationships. Colleagues not involved in the study (*n* = 8) pretested items for clarity and comprehensibility.

**Table 1.** Summary of all possible perceived fitness interdependence (PFI) and conservation ethics items generated before item selection and reduction. [Target] represents the target organism. (RC) represents items that were reverse coded. All items were rated on seven-point Likert scales.

PFI item	Response scale
When [target] become more scarce, that is … (RC) When people behave kindly towards [target], that is… When people harm [target], that is … (RC) [Target] and I rise and fall together. What is beneficial to [target] is beneficial to me, and what is harmful to [target] is harmful to me. When [target] thrive, I feel good. A world without [target] would be worse for me. [Target] and I have different fates. (RC) I feel detached from [target]. (RC)	1, Very harmful to me; 4, neither harmful nor beneficial to me; 7, very beneficial to me 1, Strongly disagree; 4, neither agree nor disagree; 7, strongly agree
Conservation ethics item	Response scale
[Target] deserve to be protected from harm. [Target] should not be treated with care and compassion. (RC) [Target] deserve to be treated fairly. I have sympathy for [target]. Harming [target] is morally right. (RC) People are entitled to harm [target]. (RC) Killing [target] is morally wrong. It would be important to protect [target] from extinction. People should deny moral concern for [target]. (RC) I feel no personal sense of responsibility to help [target]. (RC) One of the worst things a person could do is to harm [target].	1, Strongly disagree; 4, neither agree nor disagree; 7, strongly agree

### Participants and procedure

Following Ayers et al. (2023), we conducted a power analysis with *α* = 0.05, 80% power and an effect size estimate of ƒ^2^ = 0.06 and determined that we needed a sample size of at least 514 respondents. We recruited 665 adults at least 18 years of age residing in the US via Qualtrics (Qualtrics, www.qualtrics.com), a respondent recruitment platform enabling researchers to access diverse samples. We minimised unreliable responses by automatically screening out respondents who failed at least one of two randomly inserted attention checks (i.e. if you are reading this, select harmful to me; if you are reading this, select agree) or completed the task in less than 100 s (half the median response time). We further excluded 10 respondents who took longer than 800 s (four times the median response time) and four respondents who provided identical responses to all survey items (i.e. straight-lining) – an indicator of low response quality (Zhang & Conrad, 2014). Our final dataset contained a total of 651 completed sets of responses (334 males, 313 females, two non-binary, one transgender, one other; age, mean, *M* = 47.73, standard deviation, SD = 16.57), with an average of 43.4 responses per target organism (minimum *n* = 37, maximum *n* = 49). Cornell University's Institutional Review Board approved this study (IRB 2110010651), and all respondents provided informed consent prior to completing the survey.

We randomly assigned each respondent to a single organism selected from a set of 15 plants and animals (Figure 2). We first asked whether respondents knew the target organism. If respondents reported that they did not know the assigned target, we provided them with a randomly selected alternative. Once they affirmed that they knew the organism, we asked them to answer two sets of questions – PFI and conservation ethics – about that organism and complete a short demographic questionnaire. First, we measured perceptions of shared outcomes with the target as degree of agreement with the nine statements in the set using seven-point Likert-type scales (i.e. very harmful to me–very beneficial to me; strongly disagree–strongly agree) (Table 1). We then measured respondents’ moral concern for the target as degree of agreement with a set of 11 statements rated on the same seven-point scale (i.e. strongly disagree–strongly agree) (Table 1). To prevent priming effects, we randomised the order of items within each set. Lastly, we measured respondents’ demographic characteristics using four factors (i.e. sex, ethnicity, age, ZIP code). Wording for all survey items is presented in the Supplementary Materials (Supplementary Table S1).

### Species selection

We selected target organisms based on results from a multidimensional study of how people in the US think about wild organisms based on 20 characteristics, such as beauty, charisma, harmfulness to humans, trophic type, familiarity and moral standing (Hare, 2018). This study found that wild organisms cluster into three distinct groups: (1) ‘plants’, (2) ‘nasty animals’ and (3) ‘nice animals’. To ensure a range of responses with regards to PFI and conservation ethics, in the current study we selected the five most familiar organisms from each cluster identified in that initial study. ‘Plants’ and ‘nice animals’ were characterised as more charismatic, of higher moral standing and more ecologically, economically and culturally valuable whereas ‘nasty animals’ were perceived significantly more as threats or pests to humans (Hare, 2018).

### Analysis

We assessed the underlying factor structure for each set of items (PFI and conservation ethics) by performing exploratory factor analyses using oblique rotation with principal axis factoring (Supplementary Figures S1 and S2). We selected the oblique rotation method based on the assumption that the extracted factors are somewhat correlated (Osborne, 2015). To determine the suitability of the data for factor analysis, we used the Keyer–Meyer–Olkin (KMO) criterion (Kaiser & Rice, 1974) and Bartlett's sphericity test (Bartlett, 1950), and used screeplots (Cattell & Vogelmann, 1977) and parallel analysis (O'Connor, 2000) to determine the number of factors to extract for data across targets. For both sets of items, screeplots suggested that one factor should be extracted whereas parallel analysis suggested two factors. We therefore considered one-, two- and three-factor (one above what parallel analysis suggests) solutions for PFI and conservation ethics data across targets. We eliminated any inadequate items (i.e. items that are low-loading or cross-loading; Costello & Osborne, 2005) by conducting a stepwise series of item removals based on factor loadings and re-running the factor analysis after each removal. We retained items with a rotated factor loading of > 0.5 (for the PFI scale) and 0.6 (for the conservation ethics scale) and excluded items with loadings below the selected thresholds. Scores ≥ 0.4 are considered stable (Guadagnoli & Velicer, 1988), and we selected the said cut-offs to account for item communality, potential cross-loading and number of items retained.

Items removed for loading < 0.5 for the two PFI factors included (using deer as an example target): ‘What is beneficial to deer is beneficial to me, and what is harmful to deer is harmful to me’, ‘I feel detached from deer’ and ‘Deer and I have different fates’ followed by ‘A world without deer would be worse for me’. Items removed for loading < 0.6 for the two conservation ethics factors included: ‘I feel no personal sense of responsibility to help deer’, ‘It would be important to protect deer from extinction’ and ‘People should deny moral concern for deer’. We determined the internal reliability (i.e. whether scale items consistently measure the same concept) of the extracted factors after item reduction using Cronbach's *α* (Cronbach, 1951) and McDonald's omega (McDonald, 1999). We conducted all analyses in R v4.2.1 (R Core Team, 2022) using the psych (Revelle, 2022) and GPArotation (Bernaards & Jennrich, 2005) packages.

## Results

For the reduced PFI and conservation ethics scales, two factors explained the data across targets with five and eight items, respectively. Indeed, fit statistics for both sets of items prior to item reduction indicated that the two-factor solution was the most appropriate fit for the data (Supplementary Table S2). The standardised root mean square residual (SRMR) (PFI 0.05; conservation ethics 0.03), root mean square of the residuals (RMSR) (PFI 0.01; conservation ethics 0.02), root mean square error of approximation (RMSEA) (PFI 0.082; conservation ethics 0.056) and Tucker–Lewis index (TLI) (PFI 0.952; conservation ethics 0.979) of the reduced scales also indicated a good fit within the recommended criteria, namely SRMR < 0.08 (Hu & Bentler, 1998), RMSR < 0.05 (Hu & Bentler, 1999), RMSEA < 0.08 (Browne & Cudeck, 1992), and TLI > 0.95 (Hu & Bentler, 1998) (Table 2).

**Table 2.** Summary of fit statistics for the two-factor solutions of the reduced perceived fitness interdependence (PFI) and conservation ethics scales across targets. Model fit statistics indicate that the two-factor solutions of the reduced PFI and conservation ethics scales are a good fit for the data (SRMR < 0.08 (Hu & Bentler, 1998); TLI > 0.95 (Hu & Bentler, 1998); RMSEA < 0.08 (Browne & Cudeck, 1992); RMSR < 0.05 (Hu & Bentler, 1999))

Scale	*χ*^2^	d.f.	*p*	SRMR	TLI	RMSEA	RMSR	90% CI
PFI Conservation ethics	5.39 39.29	1 13	<0.020 <0.001	0.05 0.03	0.952 0.979	0.082 0.056	0.01 0.02	(0.026, 0.156) (0.036, 0.076)

Abbreviations for indices: *χ*^2^, chi-square statistic for goodness-of-fit test; d.f., degrees of freedom; *p*, significance level; SRMR, standardized root mean square residual; TLI, Tucker–Lewis index; RMSEA, root mean square error of approximation; RMSR, root mean square of the residuals; 90% CI, 90% confidence interval for the RMSEA.

The data for PFI and conservation ethics were suitable for factor analysis, and their scales demonstrated good reliability. Specifically, two-factor solutions for the reduced PFI and conservation ethics scales showed acceptable sampling adequacy (PFI 0.78–0.83; conservation ethics 0.87–0.93) (KMO; Kaiser and Rice, 1974) and non-identity correlation matrices (PFI, *χ*^2^_10_ = 929.04, *p* < 0.001; conservation ethics, *χ*^2^_28_ = 2715.74, *p* = 0.000) (Bartlett's sphericity test; Bartlett, 1950), suggesting a good fit of the data for factor analysis (Table 3). The results also indicated acceptable internal reliability across targets (Cronbach's *α* (PFI 0.79; conservation ethics 0.89); McDonald's omega (PFI 0.83; conservation ethics 0.92)) based on a cut-off value of Cronbach's *α* = 0.60–0.70 (Hair et al., 2010; Nunnally, 1978) and McDonald's omega = 0.70 (Hermsen et al., 2013) (Table 3). Since Cronbach's *α* increases with the number of items (Hair et al., 2010), we expected relatively low *α*-values given that the reduced PFI and conservation ethics scales consist of only five and eight items, respectively.

**Table 3.** Summary of the Kaiser–Meyer–Olkin (KMO), Bartlett's test, Cronbach's *α* and McDonald's omega coefficients for the two-factor solutions of the reduced perceived fitness interdependence (PFI) and conservation ethics scales across targets. KMO and Bartlett's test values indicate that the data are fit for factor analysis (KMO > 0.8 (Kaiser and Rice, 1974); *p* < 0.05 (Bartlett, 1950)). Cronbach's *α* and McDonald's omega values indicate good internal reliability (Cronbach's *α* > 0.6–0.7 (Hair et al., 2010; Nunnally, 1978); McDonald's omega > 0.7 (Hermsen et al., 2013)).

		Bartlett's test			Cronbach's *α*	McDonald's omega
Scale	KMO	*χ*^2^	d.f.	*p*		
PFI	0.81 (individual items = 0.78–0.83)	929.04	10	<0.001	0.79	0.83
Conservation ethics	0.90 (individual items = 0.87–0.93)	2715.74	28	0.000	0.89	0.92

Abbreviations for indices: *χ*^2^, chi-square statistic for goodness-of-fit test; d.f., degrees of freedom; *p*, significance level.

**Figure 3.**
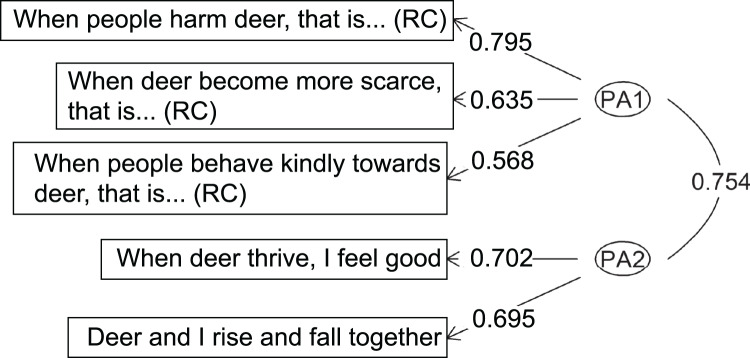
Exploratory factor structure for the two-factor solution of the reduced perceived fitness interdependence (PFI) scale across targets. Deer are used as an example target organism. (RC) represents items that were reverse-coded. Higher factor loadings indicate stronger relationships between the item and the factor. Moderate correlation between factors (~0.7) suggests that they are correlated but not redundant, and stable factor loading scores (≥ 0.4 (Guadagnoli & Velicer, 1988)) with minimal cross-loading (< 0.32 (Tabachnick & Fidell, 2001)) indicate that the two-factor solution is interpretable.

**Figure 4.**
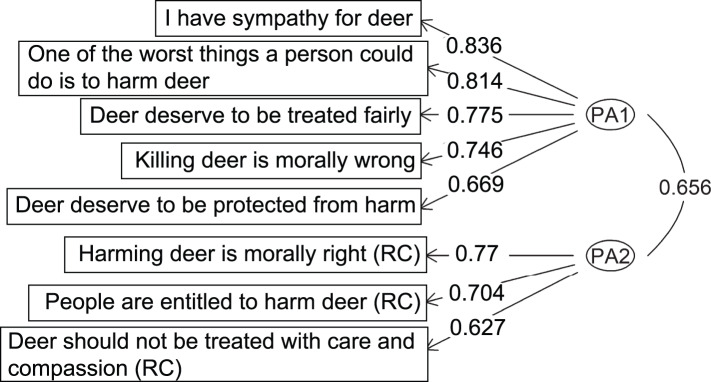
Exploratory factor structure for the two-factor solution of the reduced conservation ethics scale across targets. Deer are used as an example target organism. (RC) represents items that were reverse-coded. Higher factor loadings indicate stronger relationships between the item and the factor. Moderate correlation between factors (~0.7) suggests that they are correlated but not redundant, and stable factor loading scores (≥ 0.4 (Guadagnoli & Velicer, 1988)) with minimal cross-loading (< 0.32 (Tabachnick & Fidell, 2001)) indicate that the two-factor solution is interpretable.

For both the PFI and conservation ethics scales, the two-factor structure also emerged as the most interpretable solution (i.e. solution producing the cleanest factor structure, with item loadings > 0.3 and no or few cross-loadings; Costello & Osborne, 2005). Responses to PFI items resulted in a factor structure with one factor (PA1) identified by three items and the other factor (PA2) by two items (Figure 3, Supplementary Table S3). The factors were correlated, *r* = 0.75, and accounted for 47.8% of the total variance (explained variance per factor = 19.9–27.9%; Figure 3, Supplementary Table S3). Similarly, responses to conservation ethics items resulted in a factor structure with one factor (PA1) identified by five items and the other factor (PA2) by three items (Figure 4, Supplementary Table S4). The factors were correlated, *r* = 0.66, and explained 56.9% of the total variance in participants’ responses (explained variance per factor = 19.8–37.1%; Figure 4, Supplementary Table S4). All items loaded highest on their respective factor, and cross-loadings were all smaller than 0.24 (Supplementary Tables S3 and S4), which is < 0.32 and negligible (Tabachnick & Fidell, 2001).

## Discussion

By combining insights from evolutionary anthropology, evolutionary psychology and human behavioural ecology, we provide empirical tools to test theory about a potential cooperative basis for conservation ethics. Specifically, the PFI scale derived from a validated measure of PFI among humans (Ayers et al., 2023) contributes a novel and reliable way to index PFI with diverse wild organisms. Moreover, we show that existing individual items measuring the moral standing of humans and non-humans (Berndsen & van der Pligt, 2005; Crimston et al., 2016; Graham et al., 2011; Piazza et al., 2014) can be combined to produce a single reliable scale that measures conservation ethics.

Our finding of a two-factor solution for the PFI scale has potential implications for future investigations. Although all items contained in PA1 of the PFI scale measure perceptions of shared fate with a target, one of two items contained in PA2 measures *emotional shared fate* (Gervais & Fessler, 2017; Sznycer & Lukaszewski, 2019), and only one item explicitly measures perceived fitness responses to harming the target. Indeed, we intended to develop an internally consistent scale to measure perceptions of shared fate in general – regardless of its underlying subscales – rather than test for specific dimensions of PFI. Conversely, Ayers et al. (2023) tested and found clear support for *a priori* hypotheses regarding perceived and emotional shared fate as different but related aspects of fitness interdependence among humans. Further, the two factors of our PFI scale are highly correlated, and it may be that information provided by one factor strongly mediates the effect of the other. Future investigations of particular dimensions of interdependence, including perceived and emotional shared fate, would therefore provide valuable further insights into the nature and origin of how people perceive fitness interdependence between humans and other species.

Our novel approach also produced nuanced insights into how items measuring conservation ethics can capture subtle differences in how people think about moral concern regarding specific organisms. For example, items based on fairness, and on care and compassion loaded onto different factors and may reflect distinct domains of morality consistent with MAC (Curry, 2016; Curry et al., 2019), such as impulses promoting more equitable treatment of species based on ‘fairness’ (Curry et al., 2020), or care for companion or culturally significant species based on ‘kin values’ (Morris & Qirko, 2020; Qirko, 2017). For example, in several societies people use kin terms to describe relations with companion animals (Charles, 2014; Erikson, 2000; Rose, 2013; Wilson et al., 2013).

Items measuring moral attitudes towards harming a target were equally distributed across both factors, but we cannot assess the relationships between these items with data from this study. Future investigations of harm-related vs. other aspects of moral psychology could offer insight into the nature of human moral attitudes towards non-human organisms, such as whether emotions like ‘sympathy’ or those invoked from the ‘killing’ or ‘harming’ of species resonate with different moral rationales for conservation of those species. Although items in the current scale at face value appear to measure the same construct, our finding of a two-factor solution for conservation ethics derived from multiple existing scales indicates subtle discrepancies in how respondents think about moral concern regarding specific species. Thus, this scale provides a more comprehensive tool for measuring conservation ethics and could be adopted by future researchers interested in investigating evolutionary foundations of conservation ethics, or even to measure the moral standing of non-human species beyond evolutionary psychology.

Potential extensions of our approach would assess whether our findings from a broad sample of the US public generalise to other populations. Because different populations live in different socio-cultural and ecological environments, any extensions would have to account for relevant socio-ecologies, such as cultural beliefs and traditions as well as species composition. We conducted our study with a diverse sample of the US population using 15 common wild plants and animals (Hare, 2018). The current scales therefore promise a powerful starting point for research, particularly for investigating diverse methodological and theoretical aims in future studies (Ayers et al., 2023) such as target-specific or cross-cultural comparisons. Indeed, traditional ecological knowledge is often rooted in sustained relationships between people and ecosystems (Berkes et al., 2000) and comprises adaptations to local ecologies such as correct ways to relate to locally important species (Artelle et al., 2018; Jones et al., 2008). Recognising that traditional ecological knowledge carries important information about living sustainably in different socio-ecologies is increasingly relevant in a globalised system of biodiversity conservation (Rudd et al., 2021), as the role of Indigenous and local peoples in conserving biodiversity brings issues of equity and injustice into sharp relief (Berkes, 2017; Fletcher et al., 2021; Kashwan et al., 2021). We recognise that the current scales were developed among an English-speaking Western, educated, industrialised, rich, democratic (WEIRD) sample from a single country (Henrich et al., 2010) and are therefore relevant to that particular context. We expect PFI and conservation ethics to be sensitive to local ecological and socio-cultural conditions (Hare et al., 2018) and would encourage researchers interested in applying our scales to a different context to replicate the study in its entirety. Developing relational frameworks that are easily adaptable for use with diverse populations would promote inclusive conservation research that embraces and celebrates cultural and biological diversity, rather than expecting all people to think alike or assuming that moral psychology in the US reflects moral psychology everywhere. Replications of the entire study across multiple socio-cultural and ecological contexts could reveal the similarities and differences in relationships between PFI and conservation ethics across societies. This in turn may provide insight into how ‘rigid’ or ‘flexible’ these relationships are, and whether they vary along with characteristics of different social systems, such as population size, kinship systems, predominant livelihood types and degree of market integration (Díaz et al., 2018; Mattison et al., 2019, 2023).

Piloting enables the testing of validity and reliability of the research instruments, and feasibility of the study design, prior to data collection of the main study (Alharbi et al., 2019). In addition, following exploratory with confirmatory factor analysis on a new sample allows for testing of whether the hypothesised factor structure of a scale is consistent across different samples and thus whether it reflects its intended construct (Knekta et al., 2019; Morgado et al., 2017). The construct validity of this scale can then be assessed with convergent, discriminant, predictive and concurrent validity by incorporating other validated measures of the same construct (Morgado et al., 2017). Therefore, we acknowledge that more detailed factor structure assessment and validation of the scales would assess how robust our findings are.

We do not intend the work we present here to replace, but instead build on, long-standing proximate theories for why humans care about members of other species. Specifically, measures of PFI and conservation ethics can be combined to empirically test predictions at the ultimate-adaptive level. For example, although we did not assess in this study whether different perceptions of organisms relate to their PFI or conservation ethics, we would expect people's PFI to positively covary with moral concern towards those organisms. According to Hare et al. (2018), people are more likely to express moral responsibility towards organisms they perceive as beneficial to humans and ecosystems (i.e. plants and ‘nice animals’). Therefore, we would expect people to care more about plants and ‘nice animals’ with strong positive fitness interdependence and express more antipathy towards ‘nasty animals’ with strong negative fitness interdependence.

Explanations at different levels of analysis do not compete, and both proximate and ultimate explanations are necessary to fully evaluate the costs, benefits and constraints that shape a given behaviour (Kenrick et al., 2010; Nesse, 2019; Tinbergen, 1968; van Vugt et al., 2014). Therefore, although PFI offers a particular perspective as to why humans cooperate with members of other species at the ultimate level, it in no way represents the one and only true explanation. In fact, *domination* (Ingold, 1994; Schwartz, 2006) and *mutualism* (Wildavsky, 1991) orientation scales (Teel & Manfredo, 2010) are widely used to assess people's basic beliefs about wildlife (i.e. wildlife value orientations; Manfredo et al., 2009). Consistent with our conservation ethics index, measures of domination assess people's beliefs regarding harming or killing wildlife whereas measures of mutualism assess beliefs about caring for other species. Conversely, these measures typically target wildlife generally rather than particular species. Moreover, they commonly assess people's agreement with beliefs specifically regarding hunting and managing wildlife and the social affiliation of wildlife with humans. Empirical studies of wildlife value orientations vary widely in the types of hypotheses tested, including emergent patterns across cultures (Jacobs et al., 2022), modernisation indicators (e.g. urbanisation, income, and education) (Dietsch et al., 2016; Manfredo et al., 2019, 2016; Teel et al., 2010), socio-demographic characteristics (Bruskotter et al., 2019; Gamborg & Jensen, 2016; Teel & Manfredo, 2010) and attitudes towards wildlife-related issues and management actions (Dietsch et al., 2016; Jacobs et al., 2014; Teel & Manfredo, 2010; Teel et al., 2010). Further, studies of people's worldviews regarding conservation, such as *anthropocentrism*, *zoocentrism*, *biocentrism* and *ecocentrism* (Vucetich et al., 2015), assess their relationship with *moral inclusivity* (Batavia et al., 2020), and the acceptability of management of specific wild species (Lute et al., 2016). However, no existing measures to our knowledge have tested hypotheses at the ultimate level for why humans cooperate with different species.

## Conclusion

Our scales provide empirical, internally consistent tools for studying eco-evolutionary foundations of conservation ethics. Our study also underscores the importance of investigating both proximate and ultimate justifications for cooperation between humans and non-human organisms. Indeed, interspecific cooperation is widespread, and humans frequently cooperate with members of other species (Hare et al., 2018). Despite having only applied these scales to wildlife species prevalent in the US and to a sample of a population of a single country, the high face validity of items allows for a wide range of targets and adaptation for use across diverse cultures. We suggest our approach as a model for producing insights in other ecological and socio-cultural systems.

## Supporting information

Lee et al. supplementary material Lee et al. supplementary material

## Supplementary material

To view supplementary material for this article, please visit https://doi.org/10.1017/ehs.2024.10

## Research transparency and reproducibility

The data and R code that support the findings of this study are openly available in Figshare at https://figshare.com/s/06c21d8ed8855d17c0fa
