# Supplementary material for: Measuring perceived fitness interdependence between humans and non-humans
Source: Evol Hum Sci. 2024 Feb 27;6:e16. doi: 10.1017/ehs.2024.10 (PMC10988171; doi:10.1017/ehs.2024.10)
Supplement: Lee et al. supplementary material [file S2513843X24000100sup001.docx]

**Supplementary material for *Measuring perceived fitness interdependence between humans and non-humans***

**Supplementary Table S1.** Survey instrument for measuring respondents’ PFI, conservation ethics, and demographic characteristics. [Target] represents the target organism. All PFI and conservation ethics items were rated on seven-point Likert scales. Order of items within each block (PFI items 2–11, conservation ethics items 12–23), which included attention check questions (*) for every respondent, was randomized.

|  | Please read through this information before deciding whether to participate in our study. You may ask any questions before deciding to participate by contacting the principal researcher (details below).  By participating in this study (approximately 5-8 minutes), you will help us understand how people in the United States think about wildlife. If you choose to participate, we will ask you to respond to a series of questions about plants or animals, as well as some demographic questions.  To maintain confidentiality, we will access data using a private, password protected computer and will store your answers on a secure server. We will not ask for your name or any sensitive personal information and will not store your IP address. Only researchers directly involved in this study will be able to access the responses you provide.  Qualtrics LLC is the data controller with respect to your personal data and will determine how your personal data is used. Qualtrics LLC will share only de-identified data with Cornell University for purposes of research. We anticipate that your participation in this survey presents no greater risk than everyday use of the internet.  Participation is voluntary, and you can choose to withdraw at any point during the study. You will be compensated the amount you agreed upon before entering into the survey if you complete the study, which includes responding to some mandatory questions.  The main researcher conducting this study is Katie Lee, a graduate student in the Department of Natural Resources and the Environment at Cornell University. If you have questions about the study itself, you may contact Katie Lee at kl528@cornell.edu. If you have any questions or concerns regarding your rights as a subject in this study, you may contact the Institutional Review Board (IRB) for Human Participants at 607-255-5138 or access their website at http://www.irb.cornell.edu. You may also report your concerns or complaints anonymously through Ethicspoint online at www.hotline.cornell.edu or by calling toll free at 1-866-293-3077. Ethicspoint is an independent organization that serves as a liaison between the University and the person bringing the complaint so that anonymity can be ensured.  By confirming you are 18 and older and selecting “Yes, I agree to participate”, you indicate that you have read the above information and consent to take part in the study. | |
| --- | --- | --- |
| 1 | Do you know what [target] are? | Yes  No |
|  | On this page you will read some short statements regarding how you think about [target].  Please indicate how harmful or beneficial each of these scenarios would be for you. If you are indifferent, please select “neither harmful nor beneficial to me”.  Your responses will help us understand how people think about the impacts of wildlife on their well-being. For example, some people might consider it beneficial to have more honeybees, which pollinate plants, but harmful to have more ticks, which can spread Lyme disease. | |
| 2  3  4  5 | When [target] become more scarce, that is  When people behave kindly towards [target], that is  When people harm [target], that is  * If you are reading this, select harmful to me | Very harmful to me (1)  Harmful to me (2)  Somewhat harmful to me (3)  Neither harmful nor beneficial to me (4)  Somewhat beneficial to me (5)  Beneficial to me (6)  Very beneficial to me (7) |
|  | Next please indicate how strongly you agree or disagree with each of these statements. If you are indifferent, please select “neither agree nor disagree”. | |
| 6  7  8  9  10  11 | [Target] and I rise and fall together  What is beneficial to [target] is beneficial to me, and what is harmful to [target] is harmful to me  When [target] thrive, I feel good  A world without [target] would be worse for me  [Target] and I have different fates  I feel detached from [target] | Strongly disagree (1)  Disagree (2)  Somewhat disagree (3)  Neither agree nor disagree (4)  Somewhat agree (5)  Agree (6)  Strongly agree (7) |
|  | On this page you will read some short statements about what is right and wrong regarding how people treat [target].  Please indicate how strongly you agree or disagree with each of these statements. If you are indifferent, please select “neither agree nor disagree”. | |
| 12  13  14  15  16  17  18  19  20  21  22  23 | [Target] deserve to be protected from harm  [Target] should not be treated with care and compassion  [Target] deserve to be treated fairly  I have sympathy for [target]  Harming [target] is morally right  People are entitled to harm [target]  Killing [target] is morally wrong  It would be important to protect [target] from extinction  People should deny moral concern for [target]  I feel no sense of personal responsibility to help [target]  One of the worst things a person could do is to harm [target]  * If you are reading this, select agree | Strongly disagree (1)  Disagree (2)  Somewhat disagree (3)  Neither agree nor disagree (4)  Somewhat agree (5)  Agree (6)  Strongly agree (7) |
|  | On this page you will answer some general questions about your identity.  By responding to the following questions as accurately as you can, you will help us understand how different people think about wildlife in the US. All information you provide will be de-identified by Qualtrics and remain confidential. | |
| 24 | How would you describe your gender? | Male  Female  Other  Prefer not to answer |
| 25 | What is your ethnic background? | White  Asian  Hispanic  Black or African American  American Indian or Alaska Native  Mixed race  Other  Prefer not to answer |
| 26 | What is your age in years? | Text entry, values between 18 and 100 |
| 27 | What is your US ZIP code? | Text entry, US ZIP code |


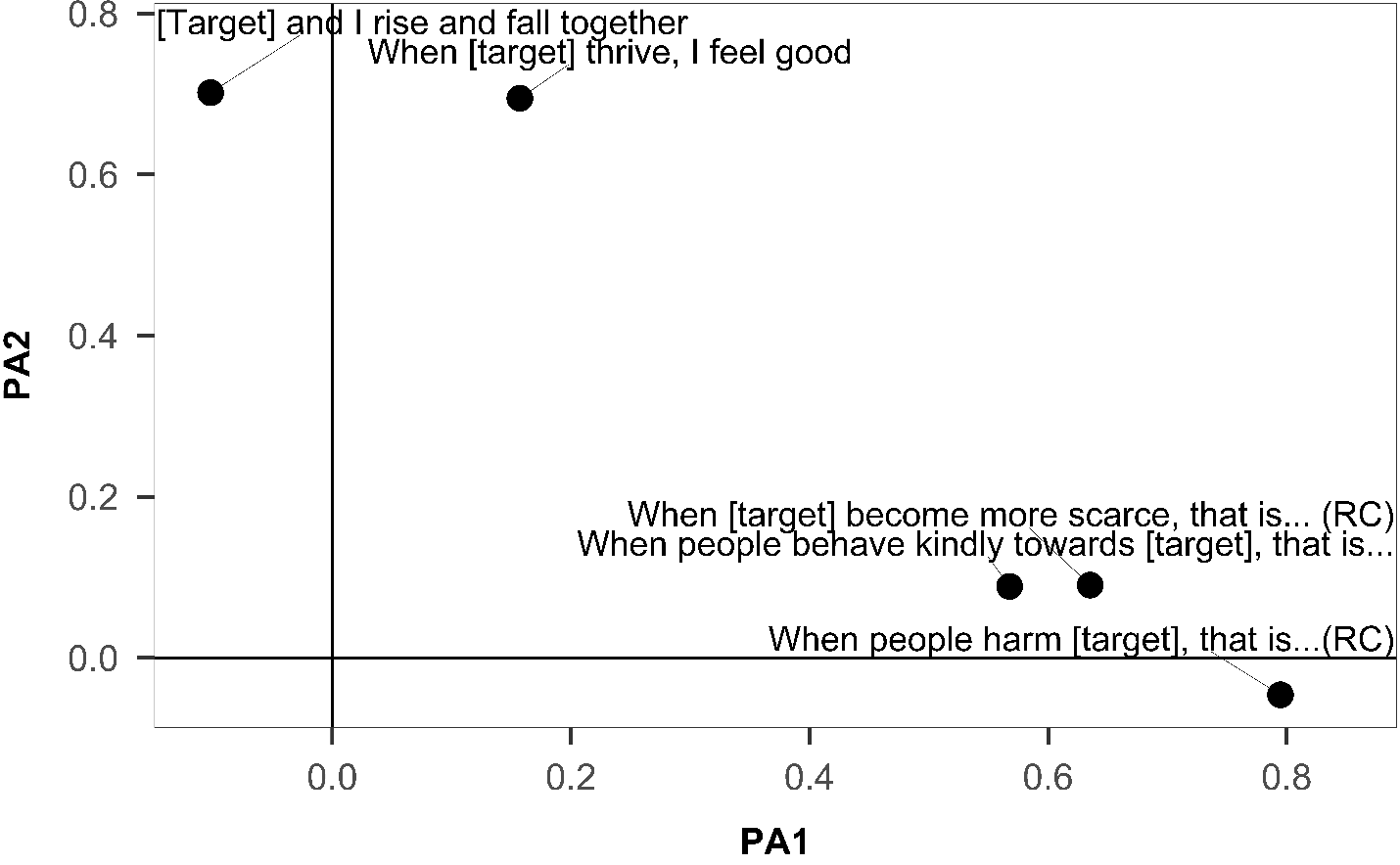


**Supplementary Figure S1.** Rotated plot of PFI item loadings across targets. [Target] represents the target organism. (RC) represents items that were reverse-coded.


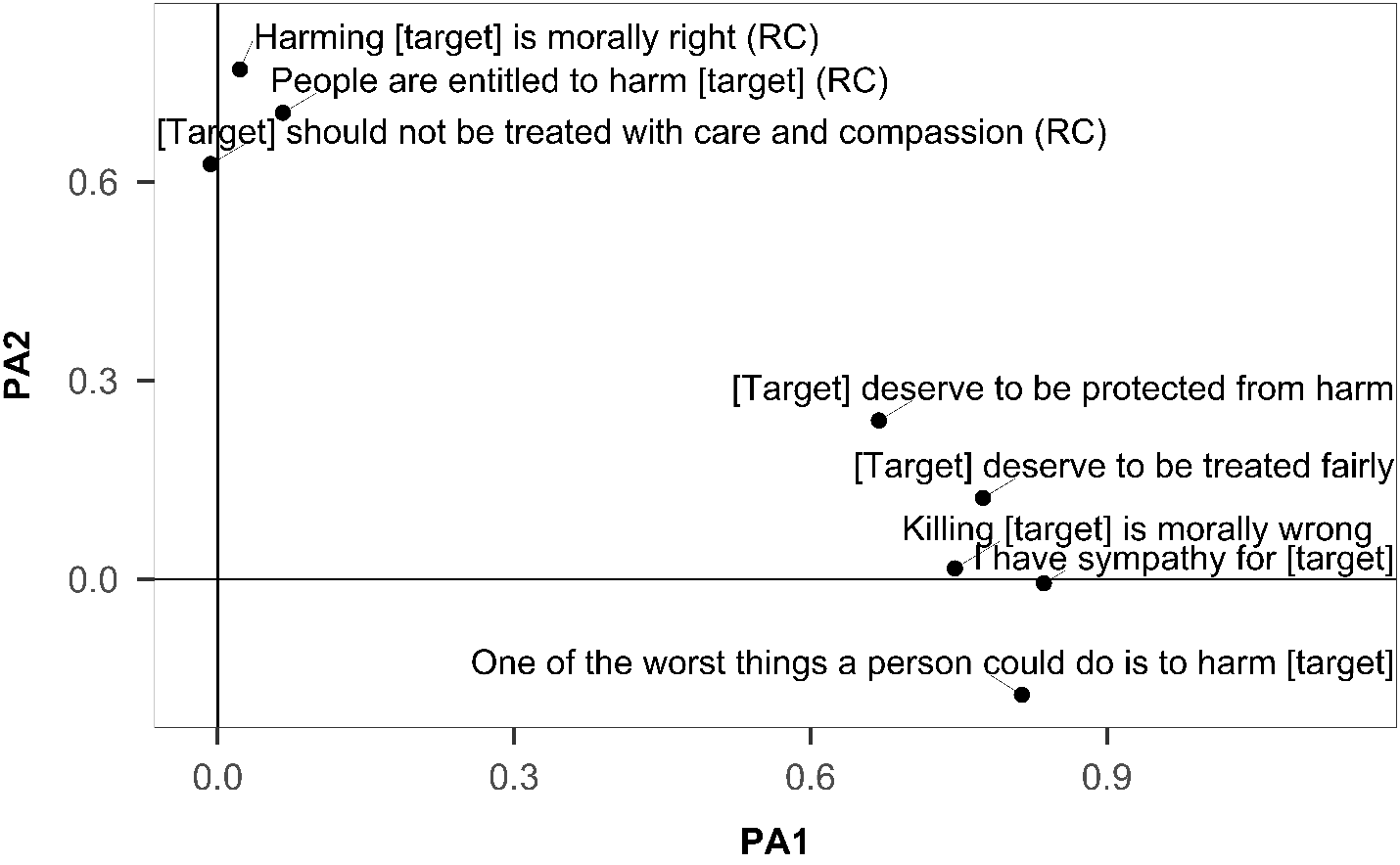


**Supplementary Figure S2.** Rotated plot of conservation ethics item loadings across targets. [Target] represents the target organism. (RC) represents items that were reverse-coded.

**Supplementary Table S2.** Summary of fit statistics for the one, two, and three-factor solutions of both sets of items–PFI and conservation ethics–prior to item reduction. Abbreviations for indices: χ^2^ = chi-square statistic for goodness-of-fit test; df = degrees of freedom; *p* = significance level; SRMR = standardized root mean square residual; TLI = Tucker-Lewis index; RMSEA = root mean square error of approximation; RMSR = root mean square of the residuals; 90% CI = 90% confidence interval for the RMSEA.

| Scale | χ^2^ | df | *p* | SRMR | TLI | RMSEA | RMSR | 90% CI |
| --- | --- | --- | --- | --- | --- | --- | --- | --- |
| PFI  One-factor  Two-factor  Three-factor  Conservation ethics  One-factor  Two-factor  Three-factor | 181.98  43.69  30.02  410.12  129.61  71.66 | 27  19  12  44  34  25 | < 0.001  < 0.001  < 0.003  < 0.001  <0.001  <0.001 | 0.07  0.03  0.03  0.07  0.03  0.03 | 0.893  0.976  0.972  0.876  0.958  0.972 | 0.094  0.045  0.048  0.113  0.066  0.054 | 0.06  0.02  0.02  0.07  0.03  0.02 | (0.081, 0.107)  (0.027, 0.062)  (0.027, 0.070)  (0.103, 0.123)  (0.054, 0.078)  (0.039, 0.068) |

**Supplementary Table S3.** Rotated factor loadings for the two-factor solution of the reduced PFI scale across targets. [Target] represents the target organism. (RC) represents items that were reverse-coded. Loadings in bold indicate the factor that items were most heavily loaded on.

| Item | PA1 | PA2 |
| --- | --- | --- |
| When people harm [target], that is… (RC)  When [target] become more scarce, that is… (RC)  When people behave kindly towards [target], that is…  [Target] and I rise and fall together.  When [target] thrive, I feel good. | **0.795**  **0.635**  **0.568**  -0.102  0.157  SS loading = 1.393  Variance explained  = 0.279 | -0.046  0.090  0.089  **0.702**  **0.695**  SS loading = 0.993  Variance explained  = 0.199 |
|  | Factors correlated *r* = 0.75 | |

**Supplementary Table S4.** Rotated factor loadings for the two-factor solution of the reduced conservation ethics scale across targets. [Target] represents the target organism. (RC) represents items that were reverse-coded. Loadings in bold indicate the factor that items were most heavily loaded on.

| Item | PA1 | PA2 |
| --- | --- | --- |
| I have sympathy for [target].  One of the worst things a person could do is to harm [target].  [Target] deserve to be treated fairly.  Killing [target] is morally wrong.  [Target] deserve to be protected from harm.  Harming [target] is morally right. (RC)  People are entitled to harm [target]. (RC)  [Target] should not be treated with care and compassion. (RC) | **0.836**  **0.814**  **0.775**  **0.746**  **0.669**  0.022  0.066  -0.007  SS loading = 2.971  Variance explained = 0.371 | -0.006  -0.175  0.123  0.016  0.240  **0.770**  **0.704**  **0.627**  SS loading = 1.585  Variance explained = 0.198 |
|  | Factors correlated *r* = 0.66 | |
